# Poleward Expansion of the White-Footed Mouse (*Peromyscus leucopus*) under Climate Change: Implications for the Spread of Lyme Disease

**DOI:** 10.1371/journal.pone.0080724

**Published:** 2013-11-18

**Authors:** Emilie Roy-Dufresne, Travis Logan, Julie A. Simon, Gail L. Chmura, Virginie Millien

**Affiliations:** 1 Department of Geography, McGill University, Montreal, Canada; 2 Redpath Museum, McGill University, Montreal, Canada; 3 Ouranos Consortium, Montreal, Canada; University of Kentucky College of Medicine, United States of America

## Abstract

The white-footed mouse (*Peromyscus leucopus)* is an important reservoir host for *Borrelia burgdorferi*, the pathogen responsible for Lyme disease, and its distribution is expanding northward. We used an Ecological Niche Factor Analysis to identify the climatic factors associated with the distribution shift of the white-footed mouse over the last 30 years at the northern edge of its range, and modeled its current and potential future (2050) distributions using the platform BIOMOD. A mild and shorter winter is favouring the northern expansion of the white-footed mouse in Québec. With more favorable winter conditions projected by 2050, the distribution range of the white-footed mouse is expected to expand further northward by 3° latitude. We also show that today in southern Québec, the occurrence of *B. burgdorferi* is associated with high probability of presence of the white-footed mouse. Changes in the distribution of the white-footed mouse will likely alter the geographical range of *B. burgdorferi* and impact the public health in northern regions that have yet to be exposed to Lyme disease.

## Introduction

Climate is a major factor constraining the niche and distribution of a species [1]. Climate is dynamic, and its influence on species is pervasive, as documented by both paleontological records and recent observations [2], [3]. Climatic conditions influence a species’ life cycle by setting the environmental conditions which affect organisms’ survival, reproduction, physiological tolerance, phenology, behavior, and sensitivity to habitat quality and food supply [4], [5]. As an effect of recent global warming, climatic fluctuations are faster and of greater amplitude than in the past [6], which further affects species’ niches and distribution patterns.

There is increasing empirical evidence that species are responding to climate warming, e.g. [3], [7]–[9], and most agree that global warming during the 20^th^ century already has had dramatic effects on the Earth’s biota [10]. Global warming challenges the stability of a species’ niche, pushing the species’ tolerance and adaptability to its limits [2]. Some species will track changing climatic conditions and shift their distribution poleward or upward in elevation, within the limit of their dispersal ability [8]. Meanwhile, climate change might increase the opportunity for invasive species to establish in new areas [11].

**Figure 1:**
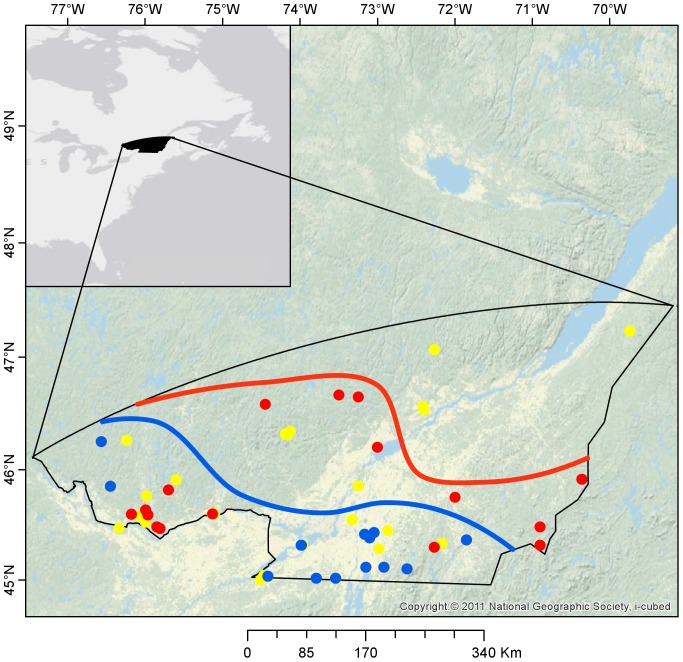
Study area for the climate niche model (ENFA). The symbols represent capture records for the white-footed mouse over its northern range coded by successive time periods corresponding to the expansion of the white-footed mouse; blue dots: 1975–1984, red dots: 1985–1994; yellow dots: 1995–2004. The lines represent the distribution limit of the mouse in 1984 (blue line) and 1994 (red line), estimated by drawing a buffer of 20 km around the presence points, using the ArcToolbox in ArcGIS [40]. Data are from the Quebec government (Ministere des Ressources Naturelles et de la Faune, Centre de donnees sur le Patrimoine Naturel du Quebec).

A prime example of a species shifting its distribution poleward is the white-footed mouse (*Peromyscus leucopus*), a successful rodent native in Eastern North America [12]. Since 1980 its population has both increased and expanded at a rate of 15 km yr^−1^ on Michigan’s Upper Peninsula [13]. Historical and recent records also document a northern expansion of the white-footed mouse in southern Québec at a rate estimated at around 10 km yr^−1^ (Figure 1). The northern expansion of the white-footed mouse is a public health concern, since the mouse is known as the main host for the black-legged tick at the larval stage (*Ixodes scapularis*), the vector for the pathogen responsible for Lyme disease (*Borrelia burgdorferi*) in North America [14].

The white-footed mouse is a generalist species and successfully occupies a wide range of habitats [15], [16]. Its distribution at the northern edge of its range is limited by a number of climatic, habitat, and anthropogenic factors. The white-footed mouse faces considerably greater seasonal variation at its northern than southern range edge, and the winter is the hardest season for this species [17]. Shift in the distribution of the white-footed mouse in the Great Lakes area has been associated with a change in snow cover, minimum temperature, and precipitation [18], [19]. Photoperiod also influences the northern limit of the white-footed mouse by regulating its reproductive system [20], [21]. Finally, the probability of occurrence of the white-footed mouse is related to the degree of habitat fragmentation and availability of food resources. The habitat must provide enough food resources during the fall to enable storage for winter reserves [22], [23]. As a territorial species only a set number of white-footed mice will live in a given patch [24], [25]. New, mature individuals need to disperse, but their movements can be hindered in less favourable habitats such as agricultural fields [26], [27].

Here, we employed species distribution modeling, a widely used technique to extrapolate and forecast species’ distributions across space and time [28]–[30], to determine some of the climatic characteristics limiting the distribution of the white-footed mouse. Then, using climate change projections, we modeled its potential future distribution under global warming. We finally tested for the relationship between the predicted current distribution of the white-footed mouse and the occurrence of *B. burgdorferi*. By describing the climatic requirements of the white-footed mouse at its northern range limit, our results will improve predictions of the future geographical occurrence of, *B. burgdorferi*, contributing to the current effort made for better understanding the pattern of emergence of Lyme disease [31], [33].

## Materials and Methods

Our aim was to characterize the climatic conditions enhancing (or limiting) the poleward expansion of the white-footed mouse. Our study area encloses most of the known range of the white-footed mouse east of –95.4°W [34], between 30.2°N in the USA and 62.6°N in northern Québec, Canada. We first used a niche model for southern Québec (45.0°N–47.4°N) to determine the climatic factors that constrain the distribution of the white-footed mouse at its northern range limit (Figure 1). We then used a set of species distribution models at a sub-continental scale, over the entire study area. This large scale was selected to maximize the predictive power of the models to project the poleward range shift [35], [36].

### Species Presence Data

Occurrence data for the white-footed mouse were obtained from the Arctos Collection Management Information System (http://arctos.database.museum, using records from the University of Alaska Museum of the North, the Museum of Southwestern Biology and the Museum of Vertebrate Zoology), the Banque de données sur les micrommamifères et les chiroptères du Québec [37], and the mammals collection database from the Field Museum of Natural History [38]. For the niche model, we used 73 records of the white-footed mouse in southern Quebec collected between1966 and 2011. For the distribution modeling we used a total of 404 records obtained for the entire study area that ranged from 1990 to 2011.

### Field sampling

We collected an additional 94 white-footed mice at 33 sites throughout southern Québec. Small mammals were sampled in forest patches from June to September 2011. At each site, 112 Sherman™ live traps were baited with a mixture of oat and peanut butter and placed at 4:00 p.m. in 4 grids of 28 (7×4) traps placed every 10 m, for one night. Trapping occurred for another two consecutive nights if no *Peromyscus* was captured on previous night. Individuals of *Peromyscus* were identified to the species level with a molecular method using species-specific primers as described by Rogic *et al*. [27]. All procedures were approved by the Ministère des Ressources Naturelles et de la Faune du Québec (SEG Permit #2011-05-15-014-00-S-F), and McGill University Animal Care Committee (AUP#5420).

At each field site, we also sampled black-legged ticks (*Ixodes scapularis*) during the spring, summer and fall of 2011, with three visits per site. Feeding ticks were sampled by inspection under the microscope of all small mammal bodies we collected in the field. Questing ticks were sampled in the vegetation using flag dragging [39]. Ticks were sampled by dragging a 1 m×1 m flannel cloth along sets of 4 parallel 500 m-long transects spaced 30 m apart, overlapping the trapping grids. Ticks were collected from the drag every 25 m along each transect. All ticks sampled were preserved in ethanol, identified to the species level and assigned to larval, nymph or adult life stages.

### Climatic Variables

A georeferenced database of climatic variables for the period 1961–2005 was generated with ArcGIS10 [40] to calibrate our models. The white-footed mouse is expected to be most limited by climate conditions during the coldest time of the year at the northern limit of its range. We used five climate variables that may constrain the distribution of the white-footed mouse and characterize the mean conditions during the winter: mean snow depth, mean precipitation, minimum and maximum temperatures, and winter length. Other factors affect the white-footed mouse’s survival (e.g., photoperiod [20], [21]), but were not considered here, as we limited our set of variables to those that were available both for current conditions and future climate scenarios, and were expected to change in the future. Photoperiod was thus not considered, as it is not a climatic variable and as a function of latitude, would not change in the future. Temperature and precipitation data were derived from the ANUSPLIN dataset version 4.3, based on Natural Resources Canada’s historical monthly ∼10×10 km gridded weather data [41]. Average monthly temperatures were centered on the Julian day of the middle of each month and linearly interpolated for the remaining days. Monthly snow depth was interpolated using data from Environment Canada meteorological stations for Québec (http://climate.weatheroffice.gc.ca) and NOAA stations for the United States (http://www.ncdc.noaa.gov/cdo-web/). Winter season was defined as the period from when the interpolated temperature of a grid cell first fell below 0°C after July, until it rose above 0°C in the following calendar year.

Future climate scenarios for the 2050 horizon (2041–2070) were created using the delta (Δ) method [42], in which the monthly mean difference of temperature, or ratio of precipitation, and snow depth between a control model run (1971–2000) and the future climate model run are calculated, then applied to baseline values of the gridded observed monthly climate data for the same control period. Future climate scenarios were created using simulated future climate data obtained from the Canadian Regional Climate Model, CRCM4 version 4.2.3 [43], as well as an ensemble of global climate simulations. Nine CRCM4 simulations were run over a domain covering North America (201×293 grid points) with a horizontal grid-size of 45 km (true at 60°N). Each run was driven by atmospheric fields simulated by one of three different coupled global climate models (CGCM3, CNRM, and ECHAM5). An additional 28 future climate scenarios were produced using output from an ensemble of global climate models (GCMs) available from phase three of the Coupled Model Intercomparison Project (CMIP3) [44]. Study requirements in terms of time horizons and variables (particularly snow depth) limited the number of available scenarios to a total of 37, divided among the Intergovernmental Panel on Climate Change (IPCC) SRES emissions scenarios (12 A1b, 15 A2, and 10 B1) [45].

### Climate Niche Modeling

We used a niche model to characterize the climatic conditions associated with the expansion of the white-footed mouse at the northern edge of its range. We performed an ecological-niche factor analysis, ENFA [46], available in the adehabitat package version 1.8.7 in R statistical software [47], [48]. This method summarizes the environmental predictors of a species’ niche with a number of factors [47]. The marginality factor describes the difference between the average conditions at the sites where the species was captured and the conditions available over the entire study area. The first specialization factor is then extracted by maximizing the ratio between the variance of environmental predictors for the global study area and the variance for the species’ distribution area. We tested the significance of these factors with randomization tests with 1,000 permutations. We also calculated the tolerance index, which is the inverse of the first specialization factor and ranges from 0 in highly specialized species to 1 in highly generalized species [47].

### Distribution Modeling

The current and future distribution of the white-footed mouse were modeled by running seven niche-based modeling techniques available in the BIOMOD platform Version 2.0.0 (BIOdiversity MODelling) [49]. We selected the artificial neural networks (ANN), classification tree analyses (CTA), generalized boosting models (GBM), generalized linear models (GLM), flexible discriminant analysis (FDA), multivariate adaptive regression splines (MARS), and random forest (RF) [49].

The quality of the predictions of the models was evaluated using a cross-validation technique. The original presence dataset was split into two subsets, with 70% of the data kept for calibration and 30% for evaluation. This procedure was repeated 10 times.

We further estimated the predictive performance of each model using the Area Under the ROC (Receiver Operating Characteristic) curve, AUC [50], and the True Skill Statistic, TSS [51]. Sensitivity (the proportion of actual presence points correctly predicted) and specificity (the proportion of actual absence points correctly predicted) were obtained for each model. The TSS maximizes the sum of the sensitivity and the specificity. Models with a TSS > 0.8 [51] and an AUC > 0.9 [50] are considered very accurate.

All models required both presence and absence data. Because our data set was assembled using mostly museum specimen databases, it did not include absence data. We thus generated five data sets of 404 random pseudo-absences using the surface range envelope (SRE) model in BIOMOD [49], with similar weight for pseudo-absence and presence points used for modeling [52]. It has been argued that pseudo-absence data introduce a bias for modeling species distribution. However, absence data could also be biased and only reflect the presence of a geographical barrier that prevents access to a site, rather than unsuitable climatic conditions [29], [30]. Here we followed the recommendations of Lobo and Tognelli [53]. They showed that the method we used to select pseudo-absences data (i.e. outside the environmental envelope obtained from the presence-data) does not affect the model performance, especially when a small number of pseudo-absence is used.

A consensus model was obtained by calculating the weighted mean of the presence probability obtained from each model [54]. Models were ranked according to their TSS score and a decay of 1.6 was used to assign relative weights to each model. Coefficients of variation between the model projection outcomes for the different runs were calculated to identify regions where these outcomes varied the most.

### *B. burgdorferi* Occurrence and the White-footed Mouse Distribution

All small mammals and ticks that were collected either in the vegetation or on small mammals were screened for the presence of *B. burgdorferi* following the method described by Ogden *et al.* [55]. *Borrelia burgdorferi* was detected by polymerase chain reaction assays of DNA extracted from small mammal tissue (heart) and ticks using a two-test PCR procedure. First, the extracted DNA was screened for *B. burgdorferi* presence with a multiplex real-time PCR targeting the 23S rRNA of *B. burgdorferi*. Positive samples were then further tested using primers for the ospA gene as reported by Bouchard *et al.* [14]. The assays were all performed at the National Microbiology Laboratory of the Public Health Agency. Sampled sites were classified as locations with confirmed presence of *B. burgdorferi* or those without. We evaluated the relationship between the probability of occurrence of the white-footed mouse we obtained from our distribution model and the occurrence of *B. burgdorferi* at our study sites with a logistic regression model using the stats package in R [48].

## Results

### Limiting Climatic Factors at the Northern Range Edge

Relatively high marginality and low tolerance obtained in the ENFA indicated that habitat selection is occurring in the white-footed mouse (Table 1). The marginality and specialization axes were significant (all *p<0.001*). The white-footed mouse is occupying an amplitude of climatic conditions three times smaller than the whole range of variation available (Table 1). The habitat of the white-footed mouse at the northern edge of its range was constrained by the winter length, presumably the mouse avoiding areas with long winters. The mean maximum and minimum temperature in the winter also contributed to marginality, indicating that the white-footed mouse tended to avoid colder habitats. A specialization for warmer minimum winter temperature was detected with a strong correlation of this variable on the specialization axis (Table 1).

**Table 1:** Summary of results of the ecological niche factor analysis (ENFA).

	Marginality	Specialization
**Tmin**	0.417	–0.811
**Tmax**	0.487	0.332
**Prec**	–0.486	0.051
**Sdm**	0.327	–0.093
**Wlen**	–0.495	–0.468
**Global**	2.32 *	3.02 * (0.311)

The correlation between the marginality and first specialization factors and each climate variables are given. The overall marginality, specialization and tolerance (in parentheses) are also shown. *: *p<0.001*.

### Current and Future Distribution of the White-footed Mouse

**Figure 2:**
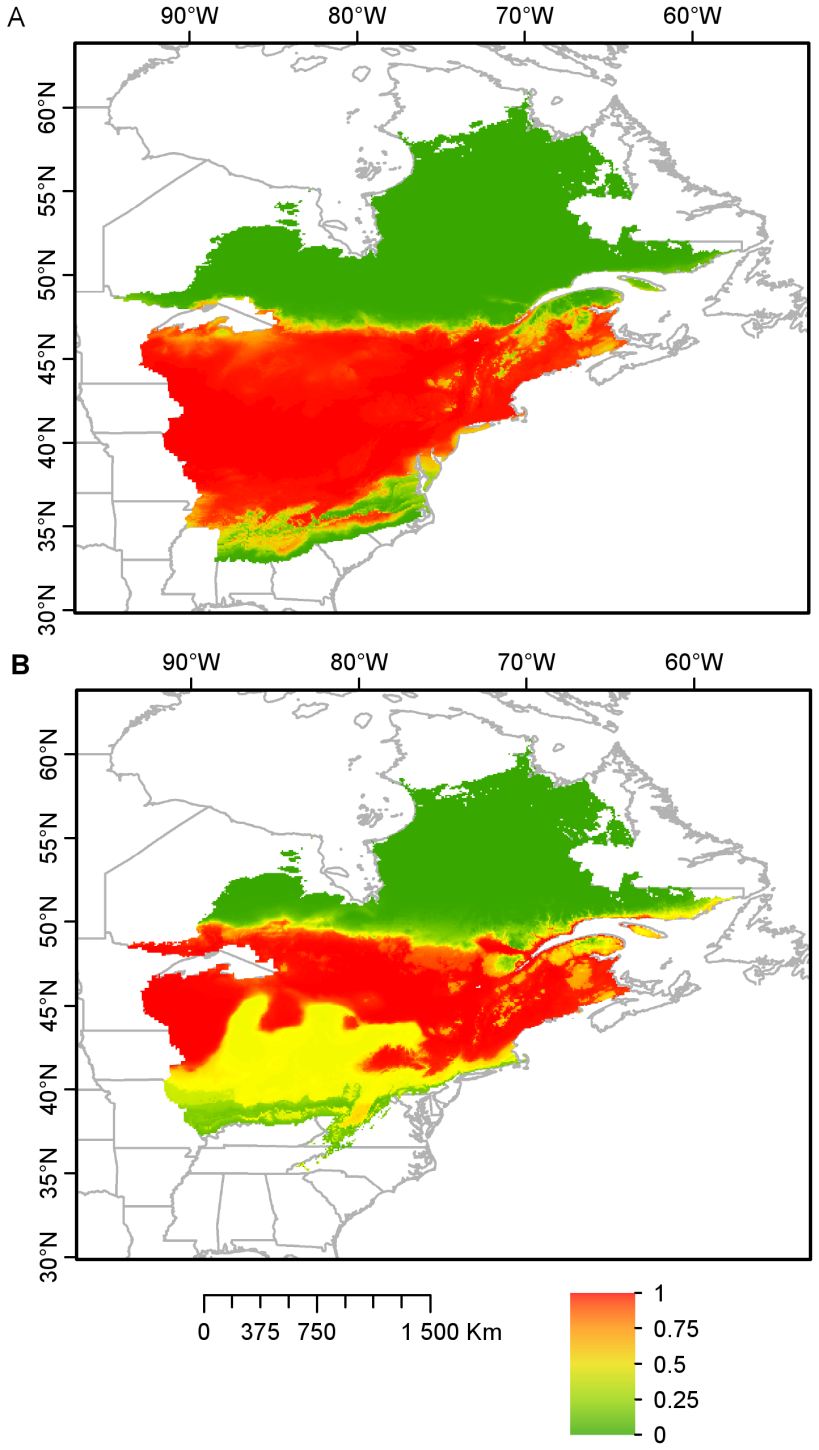
Current predicted (A) and future projected (B) distribution of the white-footed mouse modeled in BIOMOD. The projected distribution for 2050 was modeled with a change in climate under the A2 greenhouse gas emissions scenario from the IPCC [45] (WGS 1984 World Mercator). Models for gas emissions scenarios A1b and B1 are provided in Figure S1. The occurrence probability of the white-footed mouse is lowest in green and highest in red.

Overall, 75% of the entire study area had a probability of occurrence of the white-footed mouse larger than 97%, while less than 25% of the study area had a probability below 12%. The predicted probability of the white-footed mouse’s occurrence gradually decreased at the northern edge of its range, from 100% at the USA border to less than 10% north of 47°N (Figure 2A), matching well the known distribution of the species [12], [34].

Consensus models for each SRES emission scenarios produced similar projections for the future distribution of the white-footed mouse (Figure 2B, Figure S1). The probability of observing the species was greater than 94% for the majority (75%) of the study area. The probability of occurrence of the white-footed mouse decreased north, from 100% at approximately 48°N down to less than 10% at 51°N. Overall, the distribution range edge of the white-footed mouse is predicted to shift north by 3° latitude, or approximately 300 km by 2050.

The range of AUC and TSS scores were good to excellent [50], [51] for all seven models run in the BIOMOD platform, ranging from 0.71 to 0.99. The sensitivity and specificity were high for all models, ranging from 70.4% to 95.5% and from 68.9% to 93.9%, respectively.

The coefficients of variation (CV) of the model predictions ranged from 0.02 to 3.96 for the current and future (2050) consensus models. Sites with a higher CV (> 0.97 and > 0.99 to 1.6 for current and future models, respectively) represented 25% of the study area. These regions were mostly situated above 47°N, as well as in the most southern regions of our study area, where the mouse is absent today (Figure S2A). Similarly, regions where the model predictions deviated the most were located just north of the predicted northern range limit of the white-footed mouse in 2050 (Figure S2B, C and D). Overall, our model was thus better at predicting the presence than the absence, or very low probability of occurrence for the white-footed mouse.

The average winter length and maximum temperature had the greatest importance for the white-footed mouse distribution (Table 2). The probability of occurrence of the white-footed mouse decreased when winter length spanned more than 125 to 160 days, depending on the model (Figure S3). A winter average maximum temperature of –5°C represented a threshold above which the probability for the white-footed mouse to occur increased considerably (Figure S4). Winter average minimum temperature also limited the distribution of the white-footed mouse in some of the models (Table 2), but to a lesser extent. When this variable had a significant contribution to the models, the mouse’s probability of occurrence was the highest when winter average minimum temperature was below –7°C (Figure S5). Snow depth contributed to some of the models, and the probability of occurrence of the white-footed mouse decreased when snow depth exceeded 0.3 m (Figure S6). Finally, winter precipitation had no significant influence for most models (Table 2).

**Table 2:** Variable importance scores for the models used in the analysis.

	GLM	GBM	CTA	ANN	RF	MARS	FDA
**Snow**	0.136	0.008	0.125	0.008	0.090	0.058	0.040
**Winter length**	0.845	0.182	0.364	0.446	0.208	0.745	0.971
**Precipitation**	0.000	0.013	0.064	0.133	0.080	0.046	0.015
**Tmin**	0.250	0.022	0.040	0.305	0.082	0.271	0.220
**Tmax**	0.410	0.502	0.544	0.947	0.277	0.444	0.185

Snow: mean snow depth, Precipitation: mean winter precipitation, Tmin: minimum average winter temperature, Tmax: maximum average winter temperature, and Winter length: average winter length. The importance score of each variable is one minus the correlation score between the prediction obtained with all variables and the prediction obtained with only this variable. The importance score is positively related with the importance of the variable. The importance of the variables was obtained using 5 permutations for each model.

### *B. burgdorferi* Occurrence

A total of 1005 questing *I. scapularis* were collected in the vegetation (131 adults, 355 nymphs and 519 larvae) and an additional 329 feeding ticks were retrieved on small mammals (2 adults, 23 nymphs and 304 larvae). A total of 515 small mammals were collected, including 315 *P. leucopus*. Overall, 11.4% of the ticks examined (all ticks except for larvae collected by dragging) and 7 small mammals (6 *P. leucopus*) tested positive for *B. burgdorferi*. Altogether, when considering both tick and small mammal data, the bacterium was detected in 10 of our 33 field study sites sampled in southern Quebec. We found a significant relationship between of the probability of occurrence of the mouse and the occurrence of *B. burgdorferi* (logistic regression: z = 2.43, *p<0.015*). *Borrelia burgdorferi* was detected at sites where the predicted probability of presence of the white-footed mouse was above a threshold value of 97%.

## Discussion

We estimated that the expansion of the white-footed mouse into Southern Québec has been occurring at a rate of approximately 10 km yr^−1^ over the last four decades. This result is consistent with the rate of 15 km yr^−1^ observed in Michigan over the last three decades [13]. We predicted a further northern shift of 300 km over the next four decades, representing a rate of approximately 8 km yr ^−1^, a conservative value when compared to the actual rate of recent expansion of the white-footed mouse.

In our first set of analyses (ENFA), we identified the climatic variables — namely winter length and winter maximum and minimum temperature — that most constraint the niche of the white-footed mouse at its northern range limit. Under climate change, the average maximum and minimum winter temperature increased, while the winter length decreased, thus allowing the white-footed mouse to colonize new sites where the winter was warmer and shorter. Overall, climatic variables appear to describe well the habitat preference of the white-footed mouse, and thus capture the dynamics of its range shift in a changing climate.

In our second analysis, we compared the current and projected distribution of the white-footed mouse obtained from species distribution modeling. The models presented accurate predictions of the white-footed mouse’s current distribution, matching well its known distribution [12], [34]. In our models, winter length and winter average maximum temperature had the greatest influence on the distribution of the white-footed mouse, concordant with results obtained in the ENFA analysis. As future winter conditions become more clement across northern North America, the northern range limit of the white-footed mouse may extend up to 51°N in Québec by 2050.

Our results are consistent with observations of the white-footed mouse expansion into northern regions of the United States. Myers *et al*. [19] stated that the white-footed mouse would not be able to survive long winters, especially during years when ice lasts late into the spring (late April and early May). Similar observations were made in Wisconsin, where temperature and snow cover appeared to influence the species’ abundance [18]. The similarities between results from these studies in the northern United States and ours for Québec suggest that these trends are not site-dependent and that climate factors play an essential role at constraining the white-footed mouse distribution independently, to some degree, of local biotic or habitat conditions.

Our distribution model is based on the assumption that the distribution of the white-footed mouse is mostly limited by climatic factors, as it is routinely done with most work involving species distribution modeling (review in [29]). Recently, there has been a growing awareness that the distribution of a species is the result of a number of interacting factors, in addition to climatic ones [29], [56]. Range shifts in response to recent climate warming are thus modulated by other factors of the environment, such as landscape structure or the existence of strong geographic barriers [57], [58], local interactions with coexisting species such as competition [56], or the dispersal behaviour of the study species [30]. Moreover, species distribution modeling assumes no evolution of the relationship between the study species and its environment (e.g. the evolution in time of its thermal tolerance). Araujo and Peterson [56] argued that based on empirical evidence, the niche of species is conserved to some significant degree at the temporal scale considered in most studies. In sum, the predicted distribution obtained from our models should be viewed as the potential future distribution of the white-footed mouse, which does not take into account the effects of species interaction, the dispersal ability of the mouse or features of the landscape than may hinder its dispersal [30]. Yet, as Araujo and Peterson [56] noted, there is a considerable body of empirical evidence that climate plays a large role in determining species distribution. In their recent extensive study, Xu et al [59] found that the local landscape only accounted for 1–3% of species richness in terrestrial vertebrates (mammals, reptiles and amphibians) in China, while climate was identified as the factor influencing most species richness patterns. The results we present here can thus be taken as a preliminary but good estimation of the future potential distribution of the white-footed mouse in southern Quebec under climate warming.

A number of infectious diseases are emerging and their incidence has risen over the last few decades [60]. Furthermore, a majority of emerging infectious diseases are zoonotic [60]. Global change has been associated with the emergence of infectious diseases [61], [62] and species distribution projections under climate warming have become a powerful tool to better describe patterns of emergence and anticipate the future spread of infectious disease vectors, such as mosquitoes and ticks, as well as other arthropod species [63], [64]. The transmission cycle of a pathogen can be defined as a suite of species interactions that lead to target or incidental hosts [65]. Since the presence of the host constrains the transmission cycle of a disease, the geographic distribution of a disease is tied to the distributions of its hosts [63], [66].

The rapid invasion of the white-footed mouse into southern Québec has important implications for public health in the region. For a disease to emerge and spread, the disease transmission cycle must establish locally, which is ensured by the establishment of coexisting populations of the host(s) and vector(s) for that disease. In the case of Lyme disease, different hosts are used by the black-legged tick at its successive life stages. In our region, larvae, nymphs and adult ticks feed mostly on small mammals and birds foraging on the ground, mid-size carnivores and white-tailed deer [65]. Among these hosts, the white-footed mouse is known to be the most competent reservoir for *B. burgdorferi* [65], [66], and infection does not seem to affect the mouse’s behaviour [67]. The white-footed mouse is the principal reservoir host within the complex transmission cycle of *B. burgdorferi* [14], [65] and it is estimated that more than 80% of the mouse population is infected by *B. burgdorferi* in the northeastern United States [65]. We detected a significant effect of the occurrence of the white-footed mouse on the prevalence of *B. burgdorferi* in southern Quebec. Changes in the mouse distribution will inevitably alter the geographical range of Lyme disease and its prevalence [14]. This is further aggravated by the concurrent northern shift of the black-legged ticks in recent years [31], [32]. The number of established endemic populations of black-legged ticks is increasing in southern Quebec [68]. New tick populations are establishing each year, as new individuals are dispersed over the continent by migratory birds, which bring tick larvae from the southern and central United States to more northern regions, including southern Quebec [68]. There also has been an increase in abundance of the white-tailed deer in North America during the twentieth century [65]. In southern Quebec, white-tailed deer populations have exceeded historical records of abundance in the last few years [69]. As a result, both the vector (tick) and hosts (mouse and deer) are rapidly increasing in abundance in southern Quebec, which is expected to increase the encounter rate between vectors and hosts and thus provide enhanced conditions for the emergence and maintenance of *B. burgdorferi* transmission cycle in the region. Such a trend will impact public health in northern regions that have yet to be exposed to Lyme disease [33]. Our ability to predict the future distribution of the white-footed mouse is thus a critical step for identifying future areas at risk for Lyme disease.

## Supporting Information

Figure S1 **Projected future (2050) distribution of the white-footed mouse.** Change in climate variables are under the (A) A1b, and (B) B1 greenhouse gas emissions scenarios from the IPCC [45] (WGS 1984 World Mercator).
(TIF)

Figure S2 **Coefficient of variation (CV) of the current (A) and future (B-D) distribution of the white-footed mouse.** The future projections are under the A1b (B), A2 (C), and B1 (D) greenhouse gas emissions scenarios from the IPCC [45] (WGS 1984 World Mercator).
(TIF)

Figure S3 **Response curves for winter length.** Each curve represents a single run and a different graph is displayed for each model used. The y-axis is the probability of occurrence of the white-footed mouse, ranging from 0 to 1. The x-axis is winter length in days.
(TIF)

Figure S4 **Response curves for the winter maximum temperature.** Each curve represents a single run and a different graph is displayed for each model used. The y-axis is the probability of occurrence of the white-footed mouse, ranging from 0 to 1. The x-axis is the average maximum temperature in **°**C.
(TIF)

Figure S5 **Response curves for the winter minimum temperature.** Each curve represents a single run and a different graph is displayed for each model used. The y-axis is the probability of occurrence of the white-footed mouse, ranging from 0 to 1. The x-axis is the average minimum temperature in **°**C.
(TIF)

Figure S6 **Response curves for winter snow depth.** Each curve represents a single run and a different graph is displayed for each model used. The y-axis is the probability of occurrence of the white-footed mouse, ranging from 0 to 1. The x-axis is the average winter snow depth in meters.
(TIF)
