# Supplementary figures and images for: Poleward Expansion of the White-Footed Mouse (Peromyscus leucopus) under Climate Change: Implications for the Spread of Lyme Disease
Source: PLoS One. 2013 Nov 18;8(11):e80724. doi: 10.1371/journal.pone.0080724 (PMC3832455; doi:10.1371/journal.pone.0080724)

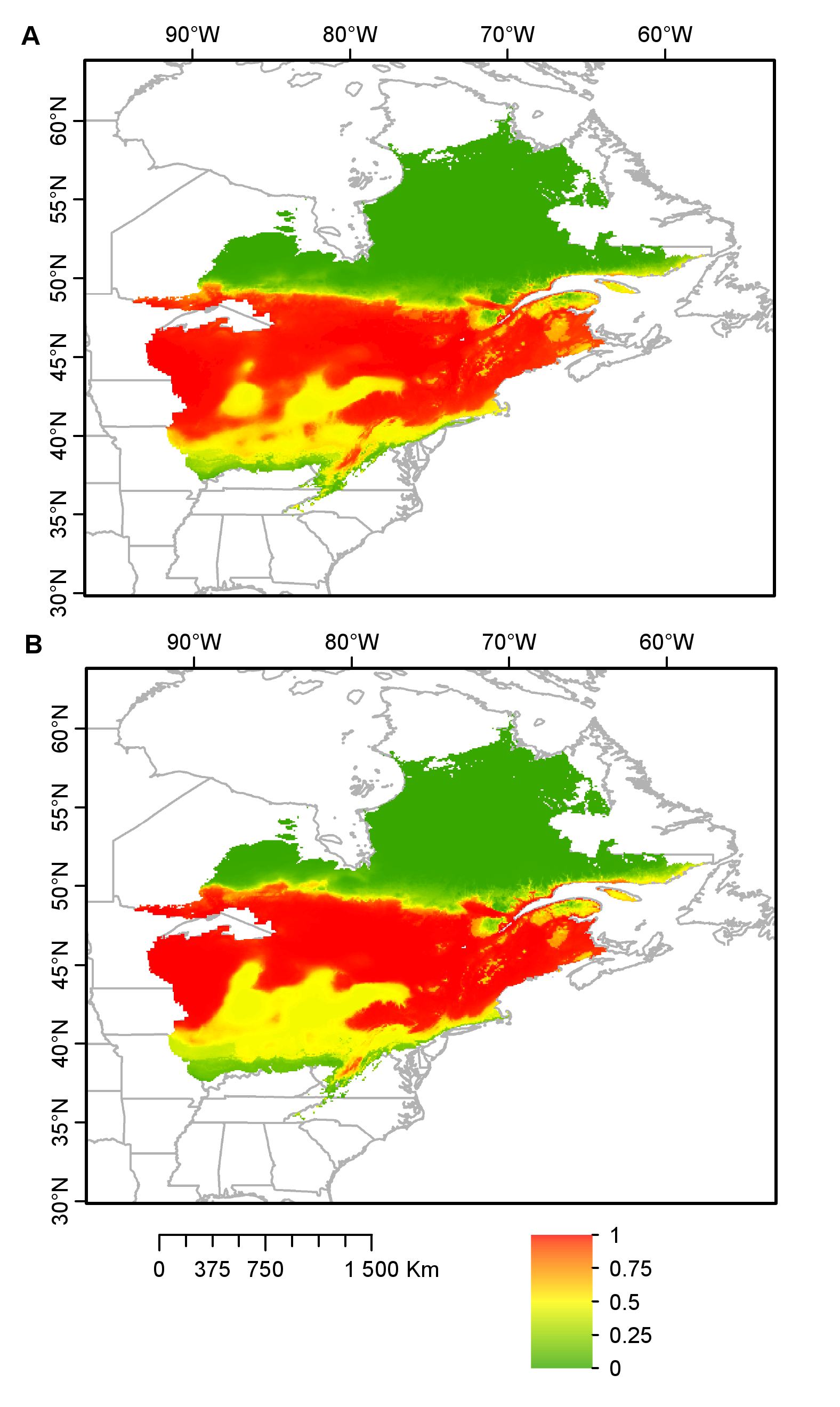

Supplement: Figure S1 — Projected future (2050) distribution of the white-footed mouse. Change in climate variables are under the (A) A1b, and (B) B1 greenhouse gas emissions scenarios from the IPCC [45] (WGS 1984 World Mercator). (TIF) [file pone.0080724.s001.tif]

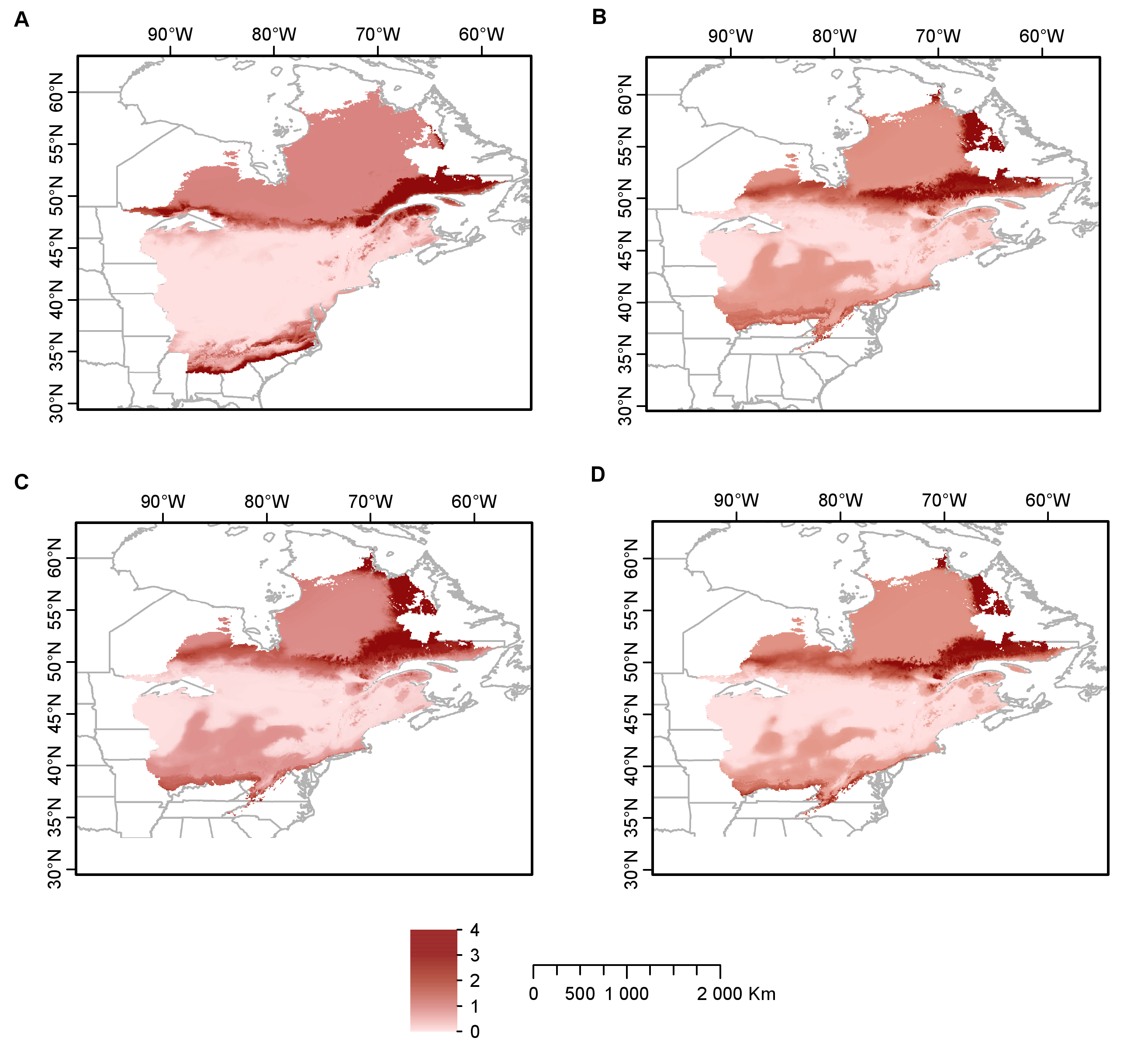

Supplement: Figure S2 — Coefficient of variation (CV) of the current (A) and future (B-D) distribution of the white-footed mouse. The future projections are under the A1b (B), A2 (C), and B1 (D) greenhouse gas emissions scenarios from the IPCC [45] (WGS 1984 World Mercator). (TIF) [file pone.0080724.s002.tif]

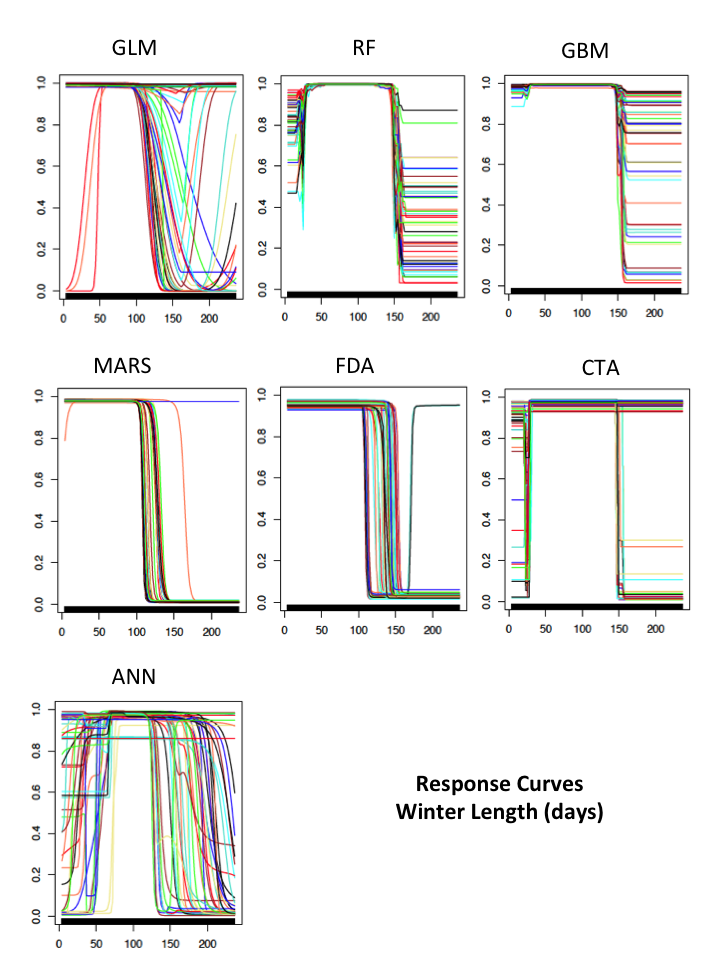

Supplement: Figure S3 — Response curves for winter length. Each curve represents a single run and a different graph is displayed for each model used. The y-axis is the probability of occurrence of the white-footed mouse, ranging from 0 to 1. The x-axis is winter length in days. (TIF) [file pone.0080724.s003.tif]

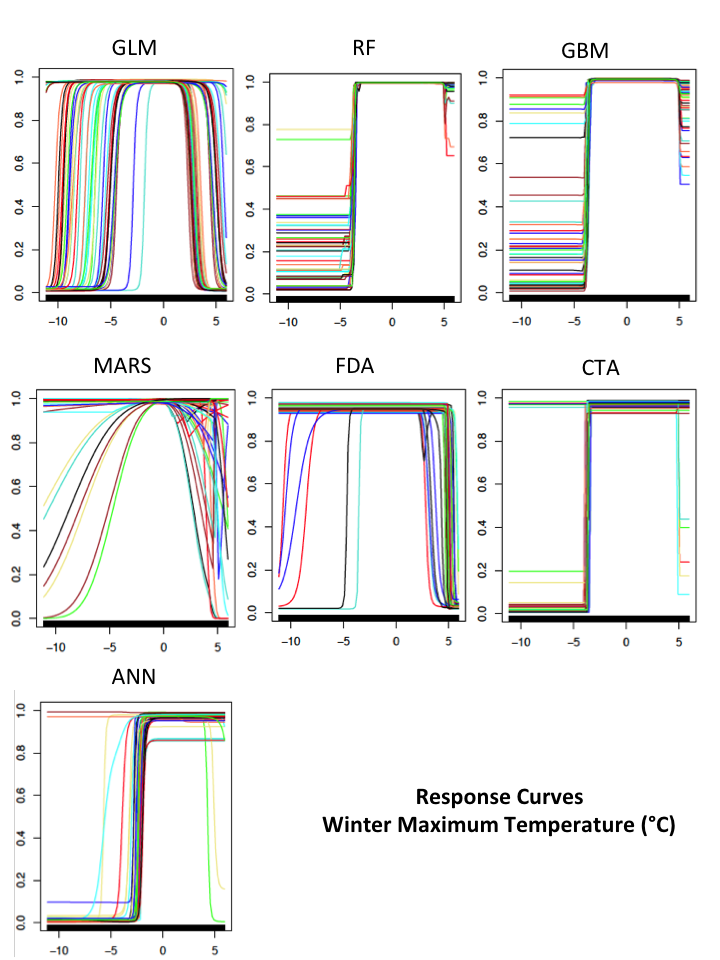

Supplement: Figure S4 — Response curves for the winter maximum temperature. Each curve represents a single run and a different graph is displayed for each model used. The y-axis is the probability of occurrence of the white-footed mouse, ranging from 0 to 1. The x-axis is the average maximum temperature in °C. (TIF) [file pone.0080724.s004.tif]

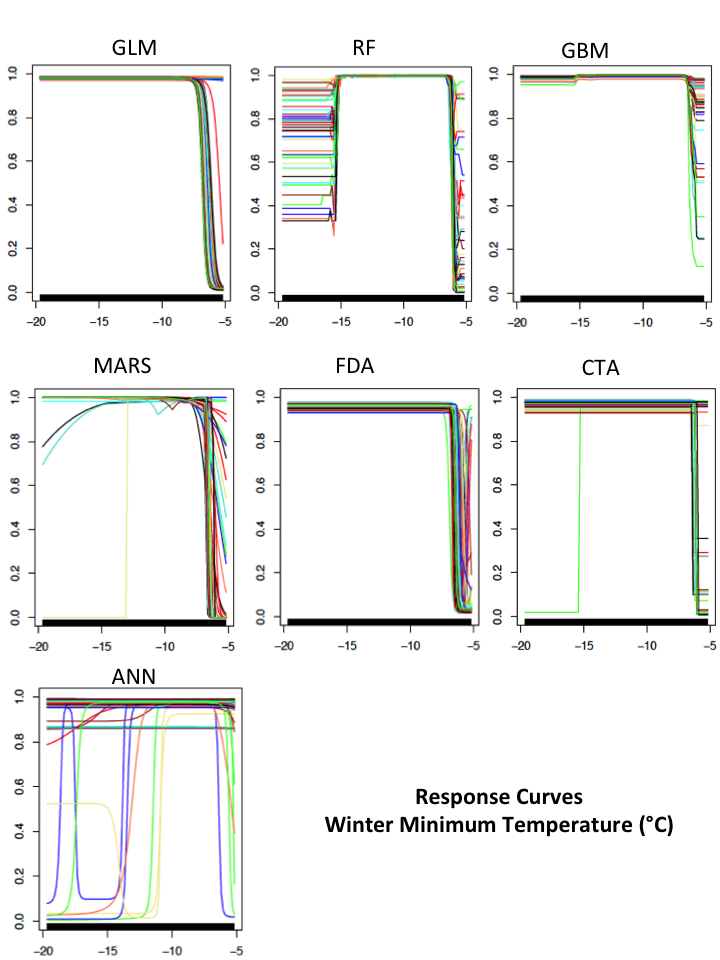

Supplement: Figure S5 — Response curves for the winter minimum temperature. Each curve represents a single run and a different graph is displayed for each model used. The y-axis is the probability of occurrence of the white-footed mouse, ranging from 0 to 1. The x-axis is the average minimum temperature in °C. (TIF) [file pone.0080724.s005.tif]

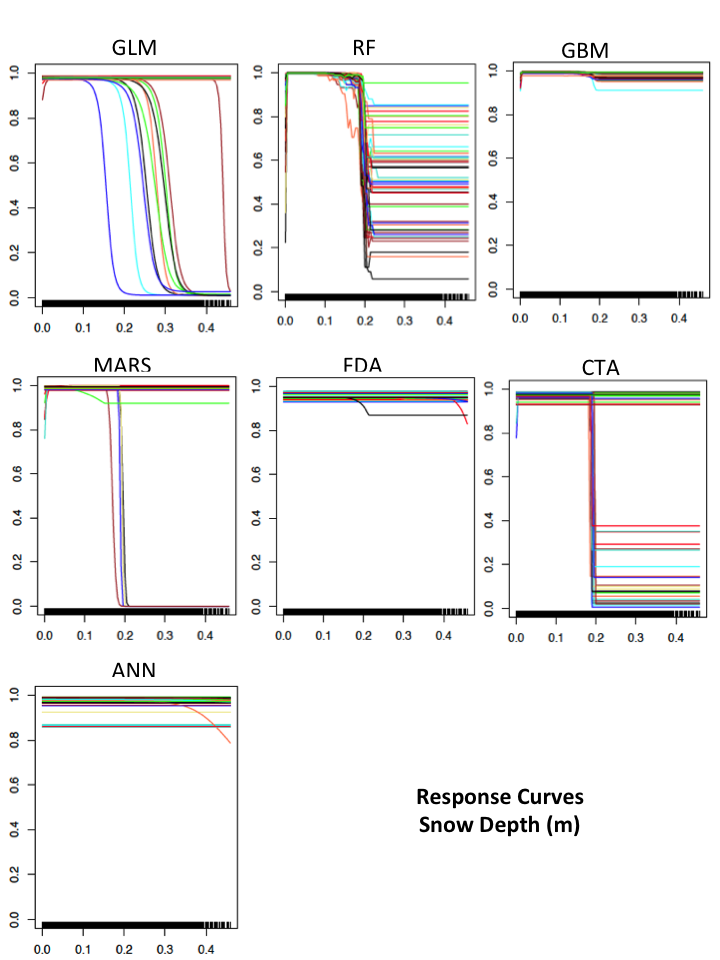

Supplement: Figure S6 — Response curves for winter snow depth. Each curve represents a single run and a different graph is displayed for each model used. The y-axis is the probability of occurrence of the white-footed mouse, ranging from 0 to 1. The x-axis is the average winter snow depth in meters. (TIF) [file pone.0080724.s006.tif]
